# A Physiological Curiosity

**Published:** 1880-03

**Authors:** 


"A PHYSIOLOGICAL CURIOSITY."—I have just received the January number of your excellent journal, and beg leave to make a few remarks on a paper by Dr. D'Ary entitled "A Physiological Curiosity"—a headless rooster. The doctor has been egregiously imposed upon. The rooster is not headless, and as good a physiologist, as the doctor doubtless is, ought to have known, when the base of the brain was disconnected from the spinal cord, the condition in which he represents his rooster, is entirely incompatible with life; it is a cruel trick, which I saw when residing in Mississippi, several years ago.

The bill of the rooster is carefully dissected away, and the skin of the neck, which is very loose, is drawn up and stitched, and finally grows, and thus the bird has the appearance of being headless. Dr. Cline, of Meridian, Mississippi, demonstrated this by performing the operation, which convinced him this was the nature of the case on seeing "a headless rooster" exhibited by some showman. He had numerous applications for such roosters, and possibly the one Dr. D'Ary saw may have been one Dr. Cline scientifically beheaded.

If you or the doctor feel further interest in the matter, or entertain any doubts, you can get full information by addressing the doctor at Meridian.—*The Physician and Snrgeon.*

